# Antiseptic effect of natural teat dip containing lactic acid against mastitis-causing *Escherichia coli*

**DOI:** 10.14202/vetworld.2019.397-401

**Published:** 2019-03-15

**Authors:** Rinrada Chotigarpa, Kannika Na Lampang, Surachai Pikulkaew, Siriporn Okonogi, Pirote Silman, Raktham Mektrirat

**Affiliations:** 1 Department of Veterinary Biosciences and Public Health, Faculty of Veterinary Medicine, Chiang Mai University, Chiang Mai 50100, Thailand; 2 Department of Food Animal Clinic, Faculty of Veterinary Medicine, Chiang Mai University, Chiang Mai 50100, Thailand; 3 Department of Pharmaceutical Sciences, Faculty of Pharmacy, Chiang Mai University, Chiang Mai 50200, Thailand; 4 Research Center of Pharmaceutical Nanotechnology, Chiang Mai University, Chiang Mai 50200, Thailand; 5 Faculty of Animal Science and Technology, Maejo University, Chiang Mai 50290, Thailand

**Keywords:** antiseptic, *Escherichia coli*, lactic acid, teat skin

## Abstract

**Aim:**

This study aimed to estimate the enumeration of total bacteria and coliform on teat skin from dairy cows and evaluate the efficacy of the natural rice gel containing 5% v/v lactic acid (NGL) against *Escherichia coli* standard and field strains isolated from bovine teat skin.

**Materials and Methods:**

A total of 100 bacterial teat skin samples (25 cows) were collected from dairy cows in smallholder farm. The cows were housed in freestall barns. The colonization of total bacteria and *E. coli* on teat skin was measured by 3M Petrifilm method. The minimum inhibitory concentration (MIC) and the minimum bactericidal concentration (MBC) of lactic acid were evaluated for reference strain of *E. coli* ATCC 25922 and two field strains of *E. coli*. The natural teat sanitizer was formulated using 5% NGL with modified rice gel. *In vitro* antiseptic efficacy of 5% NGL was determined by time-kill kinetic assay. *E. coli* morphology after exposure with 5% NGL was examined under a scanning electron microscope (SEM).

**Results:**

The total bacteria and coliform counts from bovine teat skin were 2.11×10^4^ and 1.54×10^1^ colony-forming units/ml, respectively. The MIC and MBC of lactic acid on the tested bacteria were 0.5% v/v. The natural teat dip was successfully prepared with minimum change in consistency after 1 year of storage at 4°C. The reduction rate of 5% NGL on *E. coli* ATCC 25922 and field strain showed 32.77% and 27.58%, respectively. An appearance under SEM of non-viable *E. coli* after being incubated with 5% NGL clearly showed atypical form and rough surface cell membrane.

**Conclusion:**

The rice gel containing 5% v/v lactic acid is a promising preparation as a natural teat antiseptic for reducing bacteria on teat skin. It was shown to be effective against *E. coli* causing bovine mastitis in dairy cows.

## Introduction

In the dairy industry, bovine mastitis is a critical problem of economic loss, milk loss and affects milk components. The prevalence of clinical and subclinical mastitis is 16-48% and 29-79%, respectively [1-5]. Although the prevalence of clinical mastitis is lower than subclinical, it can result in severe milk loss and a high risk of cow culling. *Escherichia coli* are the etiological agents often isolated from clinical cases in India and China [6,7]. It is normally found in environment and gastrointestinal tract of humans and animals [8]. The common sources of exposure to Gram-negative bacteria in dairy farm include manure, contaminated bedding, water, soil, and feedstuffs. It is the most frequent foodborne pathogen associated with milk or dairy products [9]. Poor milking practices are associated with the presence of extended-spectrum β-lactamase *E*. *coli* resulting in a high risk of intramammary infection and greater usage of antibiotics with the possibility of developing antibiotic resistance [10].

It is known that teat disinfection pre- and post-milking is an important tool to reduce the possibility of mastitis. Pre-milking teat disinfection will probably reduce the number of environmental pathogens, while post-milking teat disinfection is effective against contagious mastitis pathogens [11]. Pre-milking teat disinfection is not popularly used in Thailand. Hence, the prevalence of environmental mastitis pathogen is still high [12,13]. The prior study concluded that applying the pre-milking teat dipping showed a significant reduction in the number of bacteria on teat skin and new cases of mastitis caused by coliforms and environmental *Streptococcus* spp. [14,15].

2-hydroxypropanoic acid or lactic acid products have been shown to have antibacterial activities against Gram-positive and Gram-negative bacteria [16]. These ingredients are used in food and cosmetics as preservatives and also have low toxicity. From the previous report, lactic acid is less sensitive to skin irritation [17]. Many commercial teat antiseptic products have various active ingredients including iodine, hydrogen peroxide, chlorine, and chlorhexidine. Some may cause skin irritation and bacterial resistance [18]. Therefore, preparations of natural products are attractive.

The objectives of this research were to explore the number of total bacteria and coliforms on teat skin from smallholder dairy farm in Chiang Mai Province, Thailand. The antibacterial activities of 5% natural gel lactic acid (NGL) against *E. coli* were investigated by time-kill kinetic assay. The preparation of NGL affected bacterial cell structure was also observed using scanning electron microscopy (SEM).

## Materials and Methods

### Ethical approval

The study was approved by the Animal Ethics Committee of the Faculty of Veterinary Medicine, Chiang Mai University, Chiang Mai, Thailand (Approval No. S37/2559).

### Animal and study area

The study was conducted in a smallholder farm in Chiang Mai Province, Thailand, subregion of San Kamphaeng. The global positioning system of this subregion coordinates of 18°44′43″ N 99°7′13″ E. 25 Holstein Friesian cows were housed in freestall barn dairy farms and milked twice a day. Cows with good udder health were required for this study.

### Collection of teat skin samples

A total of 100 teat skin samples (1 swab/teat of 25 cows) were collected from healthy dairy cows on July 2017-August 2017. Teat skin samples were collected after washing udder with clean water and dried before the milking process using a sterile cotton swab with phosphate-buffered saline (PBS) [19]. All samples were kept at 5°C and transported to the laboratory within 4 h for total bacteria and coliform counting.

### Petrifilm method for counting bacteria

For the 3M Petrifilm method, the samples were diluted by 10-fold dilution and 1 ml inoculated on 3M Petrifilm rapid aerobic count plate and 3M Petrifilm coliform count plate for counting total bacteria and coliform, respectively. The Petrifilms were incubated at 37°C for 24 h. After incubation, the number of bacterial colonies was counted with the guideline of 3M Petrifilm interpretation. Pink colonies were enumerated as total bacteria, while red and blue colonies with gas were counted as coliform. The limitation of 3M Petrifilm rapid aerobic count plate and coliform count plate is 300 and 150 colonies, respectively. If the bacteria are too many to count, the average number of bacteria can be made by counting in one square (1 cm^2^) and multiply it by 20 to get the number of total bacteria per plate [20].

### Minimum inhibitory concentration (MIC) and minimum bactericidal concentration (MBC) determination

The bacterial strain of standard *E. coli* ATCC 25922 and the field isolates of *E. coli* were provided by the Faculty of Veterinary Medicine, Chiang Mai University, Thailand. An *in vitro* broth microdilution test was carried out to determine the MIC and MBC of lactic acid against standard *E. coli* ATCC 25922, and the field strains consisted of two unrelated isolates of *E. coli*. The bacteria cells were cultured in tryptic soy agar (Merck, Germany) plates containing 5% sheep blood, following the National Committee for Clinical Laboratory Standards recommendation. The bacterial colonies from the agar were inoculated into test tube containing Mueller-Hinton broth (Merck, Germany), and the turbidity was adjusted to 0.5 McFarland standard before use. Lactic acid was diluted into various concentrations of 10, 5, 1, 0.5, and 0.1% v/v in sterile distilled water in test tubes. A sterile 96-well plate containing equal volumes of bacterial suspension and various concentrations of lactic acid was incubated at 37°C for 24 h. Plates were observed for the absence or presence of turbidity. The minimum concentration of the lactic acid showing no turbidity was recorded as MIC value. The MBC value was determined by the drop plate technique on Mueller-Hinton agar (Merck, Germany) from the clear wells. The plates were incubated at 37°C overnight. The lowest lactic acid concentration that could completely inhibit bacterial growth is the MBC value. The MIC and MBC assays of each strain were run in duplicate.

### Preparation and characterization of natural gel containing lactic acid

The nano preparation containing 5% v/v lactic acid was obtained from the Research Center of Pharmaceutical Nanotechnology, Chiang Mai University, Thailand. The chemical modification method of milled rice grains was performed based on the method previously described by Okonogi *et al*. [21] and Chotigarpa *et al*. [22]. Briefly, the modified rice powder was used for gel base preparation and viscosity building agent. Polysorbate 80 was used for preparing an aqueous solution of lactic acid. Then, purified water was added to adjust the final concentration of lactic acid to 5% v/v. The mixture was gently stirred and then subjected to a high shear mixing device (Ultra-Turrax T25) at high-speed stirring of 5000 rpm for 1 min. The preparation was prepared under sterile condition and examined for contaminated bacteria. After freshly prepared and after 1 year storage at 4°C, the preparation was observed with physical appearances, air bubbles, color, viscosity, and pH. The pH was measured using a pH meter (Mettler Toledo, Greifensee, Switzerland).

### The time-kill kinetic assay

An *in vitro* modified time-kill analysis was performed to find out the killing time of standard and two field isolates of *E. coli*. The bacterial suspension was adjusted to 0.5 McFarland standards. An exact amount of 0.8 ml of 5% NGL and 0.1 ml of bovine fetal serum was added into 0.1 ml of each bacterial suspension. The samples were 10-fold serial diluted in PBS. The mixture samples were taken at time intervals of 30 s and 1, 5, 15, 30, and 60 min and inoculated aseptically into approximately 20 ml of plate count agar (Merck, Germany) by pour plate technique at 37°C for 24 h. Then, viable bacteria were counted. The procedure was performed in duplicate (two independent experiments) and a graph of the log colony-forming units (CFU)/ml and time were plotted.

### Detection of bacterial morphology by SEM

*E. coli* ATCC 25922 in normal saline solution (NSS) was incubated with 5% lactic acid at 37°C for 8 h, while the viable bacteria in NSS without lactic acid were used as a culture control. The treated cell was fixed using 2.5% glutaraldehyde in phosphate buffer (pH 7.4) at 4°C for 24 h. The specimen was dehydrated with graded ethanol and sputter coated with gold particles before examining the cell morphological appearance under SEM (JEOL JSM 5410LV, Tokyo, Japan).

### Statistical analysis

All tested bacteria were normalized to CFU and then log 10 transformed before analysis. The data were analyzed using one-way analysis of variance; significant differences were assumed if probabilities <0.05. Statistical analysis was performed with R statistical software (Rstudio, Boston, MA, USA).

## Results and Discussion

### Number of total bacteria and coliform count on teat skin

This study was performed during 2 months in a smallholder farm in Chiang Mai Province, Thailand. The results indicated that the number of total bacteria and coliform count on teat skin showed 2.11×10^4^ and 1.54×10^1^ CFU/ml, respectively. A higher number of total bacteria and coliform count were >2.50×10^4^ and >5.10×10^3^ CFU/ml, respectively, were reported from a previous study [23]. The differences in the sample area, environmental condition, and teat sanitation are possibly the causes for this variation [24]. The bacterial colonization on teat skin is an important source for intramammary infection [25]. Good sanitation practices can reduce the number of bacteria on teat skin and improve the milk quality, especially the pre-milking and post-milking teat dip [26,27].

### The MIC and the MBC determination

Broth microdilution methods have been used in the determination of MIC and MBC values. The MIC and MBC values of *E. coli* ATCC 25922 and *E. coli* field strains toward lactic acid were 0.5%. The MIC and MBC against all *E. coli* was 0.5% v/v. Lactic acid MBC to MIC ratios of *E.coli* was 1. This value <4 was indicated as bactericidal activity [28]. The previous results showed that the lactic acid inhibited the growth of other Gram-negative bacteria such as *Shigella* sp., *Salmonella* Enteritidis, and *Listeria monocytogenes* at 0.5% [29,30]. The other prior study reported that MIC and MBC of lactic acid against Gram-negative bacteria were ≥0.125% and ≥0.25%, respectively [31]. The difference between MIC and MBC was depended on bacteria strain tested and the concentration of lactic acid [32].

### Characterization of modified rice gel containing lactic acid preparation

The rice gel containing 5% lactic acid was successfully prepared. The formulation composed of lactic acid, rice gel, sodium chloride, and water. The property of this preparation was homogeneous, slightly air bubble, easy flow, and clear of color (Table-1). After 1 year that storage at 4°C, the pH and color of natural gel preparation were change to 2.65 and pale yellow, respectively. Topical gel is easily passed through skin, convenient to apply and more selectively to a specific site. An adhesive property is important for increasing time at the epithelium surface. The dosage form is suitable for applying in antiseptic teat dip. In addition, the topical formulation added the moisture for hydrating skin with their emollient properties [33].

**Table-1:** Characterization of a natural gel containing 5% lactic acid.

Property	5% NGL	

	Freshly prepared	After 1 year of storage at 4°C
Appearance	Homogeneous	Homogeneous
Flow	Easy	Easy
Air bubble	Slightly	None
Color	Clear	Pale yellow
pH	2.54	2.65

NGL: Natural gel lactic acid

### The time-kill kinetic assay

**Figure-1:**
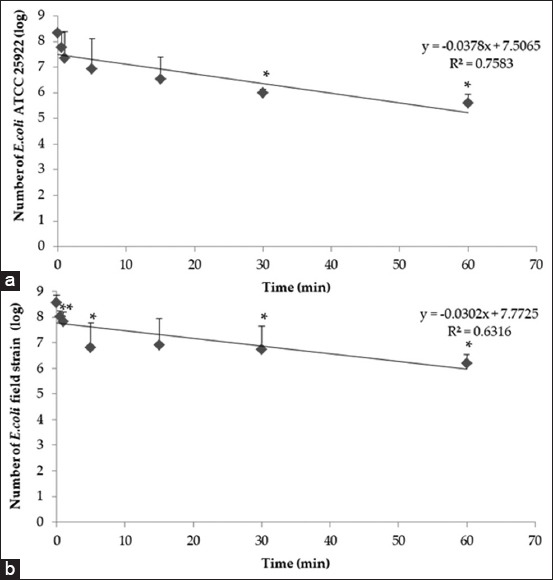
The killing rate of 5% natural gel lactic acid against (a) *Escherichia coli* ATCC 25922 and (b) *E. coli* field strain. Vertical bars represent the means±standard deviation. The line shown in the graph of the linear equation. Asterisk (*) means significant differences in bacterial count at p≤0.05.

Time-kill analysis was performed to evaluate the killing time of *E. coli* ATCC 25922 and *E. coli* field strain. The killing effect of 5% NGL was shown in Figure-1. The result demonstrated log reductions with *E. coli* ATCC 25922 and *E. coli* field strain of 3 and 2 log CFU/ml (32.77% and 27.58%) after 60 min exposure time to 5% NGL, respectively. Figure-1 shows a graph of the linear equation, with the slope of *E. coli* ATCC 25922 which had the most negative slope (k=−0.0378). The results indicated that the 5% NGL has antimicrobial ability against Gram-negative bacteria causing bovine mastitis. However, a low number of coliform were observed on teat skin (1.54×10^1^ CFU/ml). The efficiency of 5% NGL is able to against *E. coli* on teat skin bacteria. From a previous study, lactic acid has been reported to reduce the population of *E. coli* [34,35]. The incomplete killing rate may be related to the duration of exposure time.

### SEM of bacterial morphology

**Figure-2:**
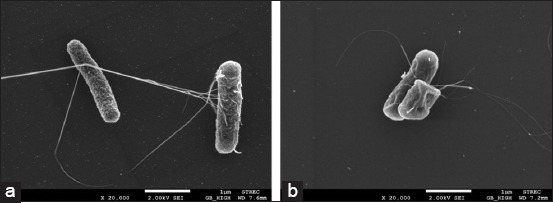
Scanning electron micrographs of *Escherichia coli* after incubation at 37°C for 8 h: (a) Viable cells (20,000×) in normal saline solution without lactic acid; (b) non-viable cells (20,000×) with 1% v/v lactic acid.

An appearance under SEM of viable *E. coli* in NSS without lactic acid as culture control is shown in Figure-2a. The untreated cells demonstrated typical rod shape with round end and smooth cell surfaces. The non-viable *E. coli* incubated with 1% v/v of lactic acid is shown in Figure-2b. After incubation with a lethal concentration of lactic acid, *E. coli* underwent significant morphological changes from rod shape to atypical form and rough surface cell membrane. Most of the studies about the mechanism of antibacterial lactic acid on Gram-negative bacteria focused on their effects on cellular membranes [16,36]. The flagella of *E. coli* are driven by the proton-motive force. From the previous study, the effect of weak acid on rotational speed of *E. coli* motors with intracellular pH was investigated [37]. When the external pH set at 5.0, the motor stopped completely. In this study, the pH of 5% NGL formulation was approximately 2.54. The impairment of flagella of *E. coli* is also found to be related to acidic pH. At acidic pH, the expression level of the flagellin-encoding gene was decreased as compared to neutral pH [38].

## Conclusion

The results of the present study show that the rice gel containing 5% v/v of lactic acid possesses antibacterial activity against *E. coli*. Our results demonstrate that the MIC and MBC of lactic acid for *E. coli* were 0.5%. The natural gel preparation did not change during storage at 4°C over a period of 1 year. The product formulation reduced the rate of *E. coli*, which means that it can play an important role in preventing new intramammary infection. It can be an alternative choice due to safe, biodegradable, and restrictions on the use of antibiotics.

## Authors’ Contributions

SO prepared the natural rice gel. RM designed the model and the computational framework. RC and RM performed the laboratory investigation. RC and KNL analyzed the data. SP and PS supported material equipment and teat skin collection. RC and RM drafted the manuscript. All authors read and approved the final manuscript.
